# The Use of Circulating Tumor Cells in T1 Stage Non-muscle Invasive Bladder Cancer: A Systematic Review and Meta-Analysis

**DOI:** 10.5152/tud.2025.24135

**Published:** 2025-04-04

**Authors:** Andy Zulfiqqar, Belinda Liliana, Nurul Lantika Mataho, Eko Subekti

**Affiliations:** 1 Division of Urology, Al Huda Hospital, Banyuwangi, Indonesia; 2 Division of Urology, IHC Kaliwates Hospital, Jember, Indonesia; 3 International Ph.D. Program in Cell Therapy and Regenerative Medicine, Taipei Medical University College of Medicine, Taipei, Taiwan; 4 School of Medicine, Faculty of Medicine Public Health, and Nursing, Universitas Gadjah Mada, Yogyakarta; 5 Division of Urology, Department of Surgery, Banyumas Regional General Hospital, Banyumas, Indonesia

**Keywords:** Circulating tumor cell, disease progression, non-muscle invasive bladder cancer, prognosis, recurrence

## Abstract

Non-muscle invasive bladder cancer (NMIBC) presents substantial variability in clinical outcomes, especially in high-grade T1 cases, which exhibit high recurrence and progression rates. Circulating tumor cells (CTCs) have emerged as a potential biomarker for cancer prognosis, with evidence linking CTC positivity to poor outcomes in various cancers. In bladder cancer (BC), studies suggest that CTC presence correlates with advanced tumor stage and treatment response, but findings are inconsistent. This study aims to clarify the association between CTC positivity and recurrence and progression to muscle invasiveness in BC.

A comprehensive search was conducted using PubMed, SciVerse Scopus, Google Scholar, and the World Health Organization International Clinical Trials Registry Platform databases up to August 2024. This study focuses on assessing the predictive ability of CTCs for NMIBC recurrence and upstaging after transurethral resection of bladder tumor (TURBT).

A total of 5 studies were included. Four of the 5 studies found a significant relationship between CTCs and recurrence after TURBT. The hazard ratio (HR) for recurrences was available in 5 studies, and the estimated pooled odds ratio (OR) predicted the value of recurrences for CTC-positive OR = 2.68 (95% CI: 2.11-3.25; *P* < .001; fixed-effect). Four studies provided data on disease progression from T1 to T2 after TURBT with an overall HR of 3.36 (95% CI: 2.68-3.25).

Circulating tumor cells enhance prognostic accuracy and therapeutic strategies in NMIBC, particularly in high-risk cases. Further studies should address molecular subtype stratification and evaluate systemic chemotherapy for CTC-positive patients.

**Main Points**

- Non-muscle-invasive bladder cancer, especially high-grade T1 cases, exhibits considerable variability in clinical outcomes, with high rates of recurrence and progression, underscoring the need for reliable biomarkers like circulating tumor cells to improve prognostic accuracy.
- The study highlights the utility of circulating tumor cell detection as a prognostic tool in non-muscle-invasive bladder cancer, particularly for high-risk patients, enhancing the accuracy of recurrence and progression predictions.
- Circulating tumor cells significantly predict recurrence and disease progression in non-muscle-invasive bladder cancer.
- The findings suggest that incorporating circulating tumor cells into clinical practice could improve treatment strategies, advocating for molecular subtype stratification and considering systemic chemotherapy for circulating tumor cell-positive non-muscle-invasive bladder cancer patients.

## Introduction

Non-muscle invasive bladder cancer (NMIBC) is characterized by its heterogeneity at both biological and clinical levels, resulting in significant variability in clinical presentation, progression, and outcomes. T1 tumors, which penetrate the lamina propria layer of the bladder, comprise about 20% of all NMIBC cases and exhibit the highest rates of recurrence, progression, and cancer-specific mortality. High-grade T1 (HGT1) bladder cancer represents the highest-risk subtype of NMIBC, associated with nearly a 40% recurrence rate and a 20% progression rate at 5 years, even after initial treatment with Bacillus Calmette-Guérin.^1^ The underlying mechanisms contributing to the aggressive nature of HGT1 bladder cancer remain incompletely understood. It is suggested that the adverse clinical outcomes observed in some patients may be due to either the understaging of T2 lesions or the inherent biological aggressiveness of the tumor.^2^ Clinical and pathological characteristics have been identified as significant factors influencing long-term outcomes.

The current treatment options for patients with bladder cancer are tailored according to the extent of cancer cell invasion into the muscle layers of the bladder, marking a shift away from the outdated “one size fits all” approach that is now considered obsolete for most other cancers.^3^ Over the past decade, 2 prognostic models developed by large European research consortia—the European Organization for Research and Treatment of Cancer and the Spanish Urological Club for Oncological Treatment—have been introduced to estimate the risk of recurrence and progression to muscle-invasive disease.^4^ These models are designed for application in the broader NMIBC population and assess risk profiles based on tumor grade and stage, previous recurrences, tumor size, multifocality, and carcinoma in situ (CIS).

Circulating tumor cells (CTCs) are tumor cells that detach from a primary tumor and circulate in the bloodstream, potentially contributing to hematogenous metastasis.^5^ The first identification of metastatic tumor cells in a patient’s peripheral blood was made by Ashworth in 1869.^6^ Detecting CTCs offers a less invasive method for early identification of metastatic disease compared to conventional methods like clinical assessments and radiographic imaging. Over recent decades, various techniques for CTC detection have been developed, including immunocytochemistry, reverse-transcriptase polymerase chain reaction (RT-PCR), flow cytometry, and the Food and Drug Administration (FDA)-approved CellSearch system.^7^ Numerous studies have indicated that the presence of CTCs serves as a poor prognostic indicator for breast, colorectal, and gastric cancers.^8^^-^^11^ However, it remains uncertain if these findings extend to urothelial cancer, which includes bladder cancer and upper tract urothelial carcinoma. Some studies have found CTC positivity to be linked with poor prognosis and associated with tumor stage and treatment response,^12^,^13^ while others have not confirmed this relationship.^14^,^15^

To further investigate this issue, a meta-analysis of published literature was conducted to evaluate the association between CTC positivity and recurrences, quantitatively including progression into muscle invasiveness of bladder cancer. Additionally, the aim was to compile and summarize the available evidence regarding the diagnostic accuracy of CTC detection in NMIBC.

## Materials and Methods

In August 2024, a systematic search was conducted (most recent inquiry: August 1, 2024) utilizing electronic repositories such as PubMed, SciVerse Scopus, Google Scholar, and the World Health Organization International Clinical Trials Registry Platform, devoid of temporal constraints. The protocol for the present systematic review followed Preferred Reporting Items for Systematic Reviews and Meta-Analyses guidelines and was registered with PROSPERO under number CRD42024601110. The employed search methodology encompassed diverse permutations of the following terminologies: “non-muscle invasive bladder cancer,” “circulating tumor cells,” “circulating urothelial cells,” “circulating bladder cancer cells,” “minimal residual disease,” “peripheral blood,” “serum,” “polymerase chain reaction,” “immunomagnetic cell enrichment,” “CellSearch,” “micrometastasis,” “urothelial cancer,” “transitional cell cancer,” “molecular staging,” and “bladder cancer cell enrichment.”

All associated publications were meticulously examined to ascertain the most significant studies and a manual review of their reference lists was conducted for supplementary relevant literature. Both primary research articles and review papers were incorporated, with the latter functioning as ancillary resources for the original investigations.

### Search Strategy and Inclusion Criteria

The current review adhered to the PICOS framework (Patient, Intervention, Comparison, Outcomes, Studies):

P—individuals diagnosed with NMIBC
I—identification of CTCs in blood specimens
C—histopathological evaluation
O—evaluative significance of CTC for recurrence and oncological progression after transurethral resection of bladder tumor (TURBT)
S—all forms of primary research studies

A systematic review of the literature was conducted using the Medline (PubMed) and Scopus databases with the search terms: (CTC OR “circulating tumor cells” OR “liquid biopsy”) AND Non-muscle invasive bladder cancer. Two authors (A.Z. and B.L.) independently reviewed titles and abstracts to exclude irrelevant publications, including reviews, commentaries, non-English articles, and studies focused on unrelated bladder cancer biomarkers or conditions.

Following this initial screening, the reviewers eliminated articles that focused solely on laboratory methods lacking clinical relevance. In cases of disagreement, A.Z., B.L., and L.M. attempted to justify their decisions and reconcile differences. If consensus could not be reached, a senior researcher (D.E.) made the final determination. The systematic review ultimately included all original research articles that explored the clinical implications of CTCs in bladder cancer diagnostics and prognostication over the past 5 years.

### Data Extraction Outcomes

Data, including patient counts, cancer stages, treatments, CTC methodologies, and marker expressions, were manually extracted from the studies. The primary focus was on assessing the predictive ability of CTCs for NMIBC recurrence and upstaging after TURBT. A meta-analysis and a qualitative narrative synthesis of the available literature were provided.

### Studies Quality Assessment

The risk of bias for each study was assessed by the Newcastle-Ottawa Scale.

## Results

**Figure 1.**
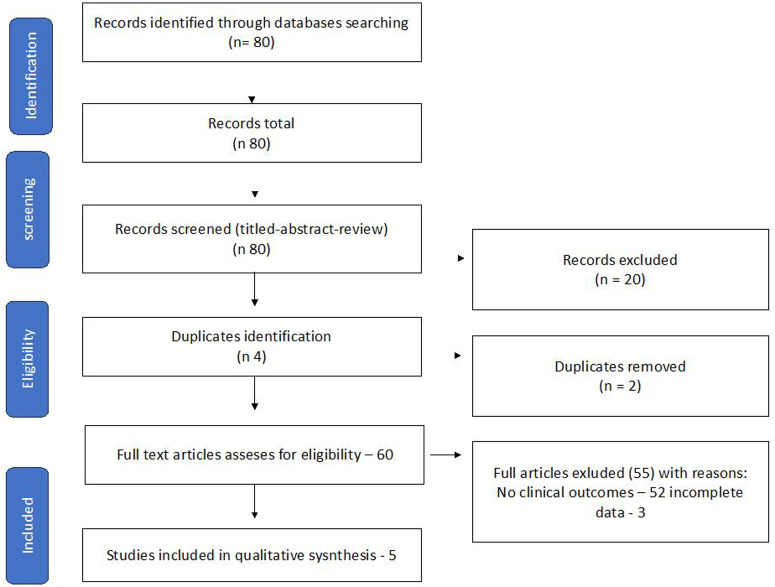
Preferred Reporting Items for Systematic Reviews and Meta-Analyses flowchart of the included studies.

Utilizing the specified search keywords, a total of 80 publication titles were identified. Of these, 6 publications were eliminated due to duplication, and an additional 20 were excluded based on their respective abstracts and titles. Consequently, 60 full-text articles were retained, then 55 studies were excluded due to no clinical outcomes and incomplete data. Ultimately, 5 studies fulfilled the inclusion criteria (Table 1). The methodology employed for study selection is illustrated in Figure 1. The evaluation of bias risk employing the Newcastle-Ottawa Scale is presented in Table 2.

1. Circulating Tumor Cell and Recurrence After Transurethral Resection of Bladder Tumor

**Figure 2.**
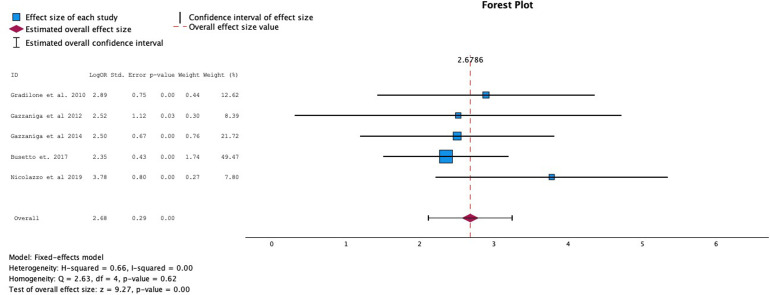
Forest plot of included studies examining the association between circulating tumor cells and recurrence after transurethral resection of bladder tumor.

Four of 5 studies found a significant relationship between CTCs and recurrence after TURBT (Figure 2), and 3 of 5 studies highlighted the multivariate analysis model that identified CTC only variable found to be statistically significant in the univariate analysis for recurrences after TURBT.^16^^-^^18^ Gazzaniga et al^19^ found that CTCs were detectable in 8 out of 44 patients, yielding an 18% detection rate in both low-risk and high-risk patients (G1, G2, and G3), with no CTCs found in 20 healthy volunteers, underscoring their potential specificity and utility in cancer detection. Another study by Gazzaniga et al^18^ and Nicolazzo et al^16^ shown CTCs were identified as strong independent prognostic factors for tumor-free recurrence (TFR) with reported hazard ratios (HR) of 2.92 (95% CI: 1.382-6.175) indicating that their presence significantly correlates with increased risks of recurrence and disease progression—surpassing traditional markers such as G status, lymph vascular invasion, and multifocality. It was found that the HR for recurrences were available in 5 studies, and the estimated pooled OR predicted value of recurrences for CTC-positive OR = 2.68 (95% CI: 2.11-3.25; *P* < .001; fixed-effect).

2. Circulating Tumor Cell and Progression After Transurethral Resection of Bladder Tumor

**Figure 3.**
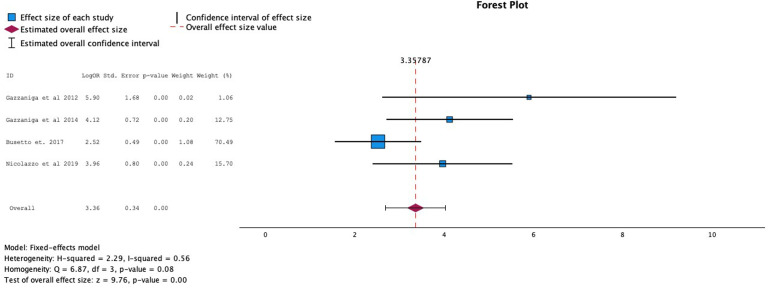
Forest plot of included studies examining the association between circulating tumor cells and progression after transurethral resection of bladder tumor.

Of all the studies included in the search, only 4 out of 5 provided data on disease progression from T1 to T2 after TURBT, with an overall HR calculated at 3.36 (95% CI: 2.68-3.25) (Figure 3).^16^,^18^^-^^20^ Gazzaniga et al^19^ conducted a multivariable Cox proportional hazards regression analysis, identifying CTC positive status as an independent prognostic factor for TFR with an HR of 2.92 (95% CI: 1.38-6.18, *P* = .005) and for time to progression with an HR of 7.17 (95% CI: 1.89-27.21).^18^ Another study also found that 87.5% of CTC-positive patients progressed to muscle-invasive disease, while none of the CTC-negative patients did.^19^ Additionally, all CTC-positive patients with local recurrence progressed to muscle-invasive disease, which correlated with the presence of CIS; CTCs were detected in 62.5% of patients with CIS compared to only 8.3% without, reinforcing CTC presence as the strongest independent predictor of disease progression.

## Discussion

Interest in blood-based genomic biomarkers for cancer diagnostics, particularly in early-stage bladder cancer, has increased due to their potential to detect residual metastatic disease after local therapy.^21^,^22^ This systematic review highlights preliminary findings on these biomarkers in managing NMIBC, a challenging condition due to inadequate prediction methods. NMIBC is complex, with most cases initially diagnosed as non-muscle invasive,^23^ yet recurrence rates can reach 78% and progression rates up to 45%, leading to cancer mortality in 16%-23% of cases within 5 years after conservative treatment.^24^

Given this high risk, NMIBC represents a key area for biomarker research to identify patients at increased risk of recurrence, progression, and death.^25^ Current prognostic predictions rely on clinicopathological parameters such as tumor grade, stage, size, multifocality, and the presence of carcinoma in situ.^3^,^26^ However, these models show poor discrimination for outcomes and low positive predictive value for progression, particularly in high-grade cases (EORTC [The European Organization for Research and Treatment of Cancer] 21%; CUETO [The Spanish Urological Club for Oncological Treatment] 24%).^26^ Thus, recent advances in molecular markers aim to address these limitations and improve outcome predictions in high-risk NMIBC patients.^27^

Circulating tumor cells represent a promising biomarker for NMIBC, primarily due to their ease of collection through blood samples and their capacity to reflect the molecular and phenotypic characteristics of primary tumors. Circulating tumor cells have been FDA-approved as prognostic indicators in metastatic cancers, including colon, breast, and prostate cancer.^28^^-^^30^ Over the past decade, 2 well-known methods for CTC identification have emerged: the FDA-approved semi-automated CellSearch® system and the manual CELLection® Dynabeads. A study by Busetto et al^20^ compared both methods, reporting comparable results. The CellSearch® system is semi-automated and quantifies CTCs, while CELLection® Dynabeads employs a more labor-intensive PCR-based approach that does not provide quantification. However, CELLection® can be combined with cell characterization tools, which is a feature that CellSearch® lacks.

Gradilone et al^17^ highlighted the superiority of CTCs over tissue-based biomarkers, particularly survivin+, in predicting recurrences in T1G3 bladder cancer. In their study, CTCs were identified as CD45−/CK8+ cells and were detected in 24 out of 54 patients, yielding an impressive 44% detection rate. Notably, CTCs were completely absent in samples from healthy controls, underscoring their potential as a more reliable and dynamic biomarker for recurrence prediction compared to traditional tissue-based methods. This finding suggests that CTCs may provide a more accurate assessment of tumor activity and prognosis in bladder cancer patients.

Circulating tumor cell clusters are emerging as critical factors in the prognosis and diagnosis of metastatic processes. However, the coexistence of CTC clusters with single CTCs in the bloodstream of cancer patients necessitates improved methods for the specific isolation of CTC clusters to enhance metastasis detection.^31^,^32^ The transition of CTC clusters through narrow blood vessels is facilitated by the cleavage of intercellular adhesions, allowing clusters to unfold into single-file chains.^33^ This rapid and reversible unfolding process enables CTCs to move as individual entities, thereby reducing their resistance to blood flow.^32^,^33^ This study’s findings indicate that CTCs found in the bloodstream during the early stages of NMIBC are associated with a worse prognosis for recurrence (OR: 2.68; 95% CI: 2.11-3.25; *P* < .001; fixed-effect) and progression to muscle-invasive disease (HR: 3.36; 95% CI: 2.68-3.25; *P* = .005). These findings suggest that certain patients may benefit from undergoing more invasive therapy in the early stages of their disease, highlighting the importance of a more individualized approach to treatment.

Furthermore, molecular characterization of CTCs is vital for correlating gene mutations with tumor progression and identifying therapeutic targets for personalized treatments. Recent research assessing genomic similarities between pulmonary venous CTCs (PV-CTCs) from surgical resections, primary tumors, and metastatic tumors has indicated that CTCs act as micrometastases associated with tumor recurrence.^34^ This underscores the importance of CTCs and their clusters in understanding cancer dynamics and improving treatment strategies.

The current approach to chemotherapy for NMIBC primarily involves intravesical chemotherapy or immunotherapy after TURBT. However, findings from studies, including 1 by Nayyar et al^35^ indicate that tumor cells can be disseminated into the circulation during TURBT, particularly in patients with high-grade and muscle-invasive disease. This raises important questions regarding the long-term oncological impact of such dissemination, which remains to be confirmed. Additionally, the predominance of muscle-invasive patients in this study population may lead to potential false positives regarding the presence of CTCs. Given these considerations, it is essential to reevaluate treatment strategies for CTC-positive patients. Specifically, this raises the question of whether the failure of intravesical therapy is linked to the presence of CTCs, suggesting a possible need for systemic chemotherapy in this population. Addressing these issues could reveal new challenges in the management of NMIBC and highlight the necessity for tailored treatment approaches.

Existing literature on CTCs in NMIBC is limited but suggests that CTCs serve as a negative prognostic factor. However, significant limitations exist in the current research. Most studies involve small sample sizes, which hinders accurate evaluation of predictive capabilities and therapeutic responses. Additionally, the efficacy of the methodologies used has yet to be established, and large prospective trials are necessary to further assess how CTCs can reliably predict tumor behavior and guide targeted therapies. Importantly, current studies have not stratified patients by molecular subtypes, complicating comparisons between individuals with seemingly similar tumors. This lack of stratification may obscure the potential for tailored treatment approaches based on specific tumor characteristics.

In conclusion, CTCs present a promising biomarker for improving prognostic predictions and treatment strategies in NMIBC. Current prognostic models based on clinicopathological parameters are limited, particularly in high-risk cases, underscoring the need for more reliable indicators. Circulating tumor cells reflect the molecular characteristics of primary tumors and may indicate tumor activity and recurrence, but existing studies are constrained by small sample sizes and a lack of stratification by molecular subtypes. The dissemination of tumor cells during TURBT raises important questions about the implications for intravesical therapies, suggesting a need for reevaluating treatment strategies in CTC-positive patients, possibly including systemic chemotherapy.

## Figures and Tables

**Table 1.** Characteristics of Included Studies

No.	Author, Year	Country	Design	Sample Size	BCG or Adjuvant Chemotherapy	Follow-Up	Detection Rate	HR (Recurrence) 95% CI	HR (Progression) 95% CI
1.	Gazzaniga et al (2012)	Italy	Prospective cohort	44 patients with NMIBC and 20 healthy volunteers	No data provided	24 months	CTCs were detectable in 8/44 patients (18%), (mean number 1.5, range 1-3) and in 0/20 healthy volunteers.	TFR Median TFR was found significantly shorter in the group of CTC+ compared with CTC− (6.5 vs 21.7 months, *P* < .001)	TTP Median TTP was not reached, due to the short follow-up period.
2.	Gazzaniga et al (2014)	Italy	Prospective cohort	102 patients with a primary diagnosis of T1G3 NMIBC	Adjuvant BCG immunotherapy scheme was started 2 weeks after the second TURB, starting with induction and followed by maintenance (instillation once weekly for 6 weeks, followed by 3 years of maintenance instillation with 3 weekly instillations every 3 or 6 months).	Median follow-up was 24.3 months (range 4-36)	Before TURB, CTCs were found in 20/102 (20%) patients. The median CTC number was 1 (range: 1-50).	TFR Multivariate HR 2.922 (95% CI: 1.382-6.175), *P* = .005	TTP Multivariate HR 7.169 (95% CI: 1.889-27.210), *P* = .004
3.	Busetto et al (2017)	Italy	Prospective cohort	155 patients with a pathologically confirmed diagnosis of high-risk NMIBC (pT1G3 with or without CIS) 101 patients’ samples (Group A) were analyzed with the CellSearch® automated system and 54 patients’ samples (Group B) were analyzed with the CELLection® Dynabeads manual system	BCG immunotherapy was started 2 weeks after the second TURB with induction plus maintenance (weekly instillation for the first 6 weeks and then 3 weekly instillations every 3 to 6 months for the following 3 years).	28 months	20 positive CTC patients in Group A (19.8%) and 24 in Group B (44.4%) for a total of 44 (28.4%).	**GROUP A** Recurrences rate 15/20 samples (75%) of CTC+ 26/81 samples (32%) of CTC− *P* < .0001 **GROUP B** Recurrences rate 20/24 samples (83%) of CTC+ 4/30 samples (13%) of CTC− *P* < .0001 TFR Multivariate HR 13.79 (95% CI: 4.47-42.56), *P* < .0001	**GROUP A** Progressions rate 13/20 samples (65%) of CTC+ 5/30 samples (6%) of CTC− *P* < .0001 **GROUP B** Recurrences rate 7/24 samples (29%) of CTC+ 2/30 samples (6%) of CTC− *P *= .02
4.	Nicolazzo et al (2019)	Italy	Prospective cohort	102 patients with HGT1 bladder cancer	BCG induction plus maintenance 1-3 years.	Median follow-up was 63 months (range 11-90)	CTCs were found in 20/102 (20%) patients. The median CTC number was 1 (range 1-50).	TFR Multivariate HR 4.26 (95% CI: 2.13-8.52), *P* < .0001 TSR Multivariate HR 3.53 (95% CI: 1.81-6.87), *P* < .0001	TTP Multivariate HR 7.32 (95% CI: 3.29-16.27), *P* < .0001
															MFS Multivariate HR 18.75 (95% CI: 6.01-58.47), *P* < .0001 OS Multivariate HR 3.68 (95% CI: 1.81-7.51), *P* < .0001 CSS Multivariate HR 13.68 (95% CI: 4.27-43.80), *P* < .0001	
5.	Gradilone et al (2010)	Italy	Prospective cohort	54 patients with T1G3 NMIBC	After transurethral resection (TUR), patients all had instillations of BCG, the schedule comprising a 6-week induction followed by 3-week maintenance (once a week for 3 weeks). The 3-week maintenance was repeated at 6, 12, 18, and 24 months.	24 months	CTCs (CD45–/CK8+ cells) were found in 24/54 patients (44%)	Mean DFS Multivariate CTC (+) 10.46 months (95% CI: 6.84-14.97) CTC (−) 23.86 months (95% CI: 23.69-24.06) *P* < .0001 Risk of relapse Multivariate OR 16.64 (95% CI: 3.572-77.46), *P* < .0001	

BCG, Bacillus Calmette-Guérin; CSS, cancer-specific survival; CTC, circulating tumor cells; DFS, disease-free survival; HR, hazard ratio; MFS, metastasis-free survival; NMIBC, non-muscle-invasive bladder cancer; OS, overall survival; TFR, time to first recurrence; TTP, time to progression; TURB, transurethral resection of the bladder.

**Table 2.** Risk of Bias Assessment Using The Newcastle-Ottawa Scale

Cohort Study	Selection				Comparability		Outcome			
	Representativeness of the Exposed Cohort	Selection of the Non-Exposed Cohort	Ascertainment of Exposure	Demonstration That Outcome of Interest Was Not Present at the Start of Study	Main Factor	Additional Factor	Assessment of Outcome	Was Follow-Up Long Enough for Outcomes to Occur	Adequacy of Follow Up of Cohorts	Total (0-9)
Gazzaniga et al (2012)	1	1	1	1	1	0	1	1	1	8
Gazzaniga et al (2014)	1	0	1	1	1	0	1	1	1	7
Busetto et al (2017)	1	0	1	1	1	0	1	1	1	7
Nicolazzo et al (2019)	1	0	1	1	1	1	1	1	1	8
Gradilone et al (2010)	1	0	1	1	1	1	1	1	1	8

## Data Availability

N/A.
